# Assessment of One Health Initiatives from a Veterinary Public Health Approach in Latin America and the Caribbean

**DOI:** 10.3390/tropicalmed10110315

**Published:** 2025-11-06

**Authors:** Baldomero Molina-Flores, Marco Antonio Natal Vigilato, Felipe Rocha, Ottorino Cossivi, Margarita Corrales, Germán Andrés Vásquez Niño, Álvaro A. Faccini-Martínez, Wagner Antonio Chiba de Castro, Alexander Welker Biondo, Natalia Cediel-Becerra

**Affiliations:** 1 Pan American Center for Foot-and-Mouth Disease and Veterinary Public Health (PANAFTOSA/VPH), Pan American Health Organization (PAHO/WHO), Rio de Janeiro 25045-002, RJ, Brazil; molinab@paho.org (B.M.-F.); vigilato@paho.org (M.A.N.V.); rochafe@paho.org (F.R.); cosivio@paho.org (O.C.); corralesm@paho.org (M.C.); 2 FAO Regional Office for Latin America and the Caribbean, Santiago 7630412, Chile; german.vasqueznino@fao.org; 3 Servicio de Infectología, Hospital Militar Central, Facultad de Medicina, Bogotá 110231, Colombia; afaccini@gmail.com; 4 Latin-American Institute of Life and Nature Sciences, Federal University for Latin American Integration, Foz do Iguaçu 85870-650, PR, Brazil; wagner.castro@unila.edu.br; 5 Department of Veterinary Medicine, Federal University of Paraná, Curitiba 80035-050, PR, Brazil; abiondo@ufpr.br; 6 School of Agricultural Sciences, De La Salle University, Bogotá 11001, Colombia

**Keywords:** governance, multisectoral collaboration, one health implementation, veterinary public health, Latin America and the Caribbean

## Abstract

The Pan American Health Organization (PAHO) launched the One Health policy in September 2021. To respond to this regional policy, a baseline was generated regarding the use of One Health intersectoral actions aimed at preventing, controlling, and eliminating zoonotic, foodborne diseases and antimicrobial resistance. For this purpose, in July 2022, the Pan American Food and Mouth Disease and Veterinary Public Health Center, Pan American, Health Organization/World Health Organization (PANAFTOSA/VPH-PAHO/WHO), organized a meeting in Rio de Janeiro, Brazil, to bring together recognized public health and animal health and food safety officers from ministries of health and agriculture from nine different countries of the region: Argentina, Belize, Bolivia, Brazil, Chile, Cuba, Honduras, México, and Uruguay, including the three representatives of the Americas in the Quadripartite Panel of One Health High Level Expert Panel (OHHLEP 2021–2024). Several good practice examples and lessons learned of multisectoral communication, collaboration, coordination, and capacity building regarding control and prevention of zoonoses, food safety, and antimicrobial resistance programs were identified in these countries. The establishment of governance mechanisms and legal frameworks were the main aspects discussed, followed by the importance of the environmental sector, which often is poorly articulated in One Health initiatives. The leadership of PAHO for more than seven decades is part of the good health governance practices to create the ground for the One Health implementation in Latin America and the Caribbean.

## 1. Introduction

The term Veterinary Public Health was first used by the World Health Organization (WHO) in 1946 to present a programmatic framework that included all public health activities related to veterinary medicine in the context of protecting and improving human health [1]. The Pan American Health Organization’s Veterinary Public Health Unit supports intersectoral actions with technical excellence to achieve measurable positive impacts on the burden of zoonotic illnesses and food-borne diseases. At the same time, it struggles with social inequality, poverty, and environmental challenges within the region. Latin America and the Caribbean (LAC) have developed knowledge, best practices and joint learning on the multisectoral and transdisciplinary work required in the prevention, surveillance, and control of zoonoses, food hygiene, and antimicrobial resistance [2,3]. Studies regarding One Health in countries of Latin America and the Caribbean have shown that most people are familiar with One Health, and many are aware of the intersectoral cooperation processes developed in their countries, mainly aimed at zoonotic diseases such as food-borne diseases, vector-borne diseases, emerging viral diseases, and neglected parasitic diseases [3,4,5]. Challenging topics to address in One Health included governance, research, integration of the environmental component, and lack of conjoint legal and funding frameworks [3,4]. However, this historical accumulation of knowledge has not been identified or defined as One Health, although it has clearly applied the 4 C’s: communication, collaboration, coordination, and capacity building, terms recognized in the new One Health definition as key elements for One Health implementation [6].

In 2021, during the COVID-19 pandemic, PAHO launched its One Health policy during the 59th Directing Council and the 73rd session of the Regional Committee of WHO for the Americas. This policy on existing mandates and plans, and on the experience of PAHO and other relevant organizations in driving positive health outcomes by working with stakeholders beyond the health sector. The PAHO One Health policy intends to provide guidance to Member States and the Bureau on best practices and strategic and systematized governance frameworks that can be adopted, adapted, and implemented by the countries of the region, considering national contexts, needs, and priorities, and supported by technical cooperation [2].

This study aims to develop a baseline for Latin America and the Caribbean, focusing on One Health intersectoral actions, providing good examples of multisectoral communication, collaboration, coordination, and capacity building in zoonosis control, food safety, and antimicrobial resistance programs that can be identified in several selected countries.

## 2. Materials and Methods

On 12 and 13 July 2022, the One Health Workshop from the Veterinary Public Health approach was held in Rio de Janeiro, Brazil, with representatives of the Ministries of Health and Agriculture of nine (9) countries: Argentina, Belize, Bolivia, Brazil, Chile, Cuba, Honduras, Mexico, and Uruguay. Countries were selected looking for their geographical representation of the four American subregions: North, Central, South, and Caribbean.

Stakeholders who joined the workshop were the focal points or responsible officers for different programs (like rabies, food safety, and brucellosis, etc.) at the country level with whom PAHO/PANAFTOSA cooperates.

Previous to the meeting, the organizers prepared a set of questions to trigger conversation with and between participants to identify the practices that create better intersectoral communication, collaboration, coordination, and capacity building in their programs. During the meeting, participants presented and described their experiences and the knowledge gained during the program activities in previous years. The facilitation team used an adapted participatory appraisal approach to collect data and foster dialog among participants. The questions used to initiate dialog based on the presentations were as follows: 1. To what extent do countries actually cooperate and create synergies between sectors in technical cooperation programs for zoonoses prevention, food safety, and AMR? 2. What actions or activities involving One Health are known in the region? 3. In which implementation field are they grouped (governance, resources, or partnerships)? 4. What reasons exist to optimize partnerships between sectors and stakeholders? 5. What are the most common barriers to implement? 6. Who is missing from the actions proposed so far (NGOs, environment, or civil society)? 7. How can we motivate the implementation in each country?

The facilitation team applied a reflexive thematic analysis, using the analytic phases suggested by Campbell et al. 2021 [7], considering the prevalence and keyness of the topics discussed according to the questions previously described. The phases and actions performed by facilitators to analyze data were:

- Data familiarization: real-time note-taking during country presentations and subsequent dialog among participants.
- Initial code generation: labeling and organizing relevant data segments—such as participant insights, country-specific reports, and intersectoral collaboration outcomes—were coded to capture recurrent ideas and issues. These codes were then grouped into broader themes, which were refined to ensure coherence and representation.
- Each theme was further analyzed to highlight the underlying context, specific examples, and actionable recommendations.
- Generating initial themes: writing themes and their defining properties, such as i. zoonotic diseases and ii. program actions—establishment of multisectoral coordination groups; epidemiology and surveillance; outbreak control; risk analysis; legislation update; capacity building of the workforce; and actions at the community level.
- Theme review: collapsing overlapping themes and refining codes and themes along the several presentations and discussions.
- Theme defining and naming: after sharing the initial theme list, feedback was requested from participants to have the opportunity to refine the themes by adding elements.
- Report production: a thematic report was written and shared with participants after the meeting.

This exercise was based in accordance with PAHO’s One Health policy action track 1 and taking into consideration the situational analysis recommended by the Joint Plan of Action of the Quadripartite (FAO/UNEP/WHO/WOAH) [8].

Additionally a review to identify the main stakeholders, networks, and initiatives related to One Health in Latin America and the Caribbean between 2005 and 2024, was carried out in order to enhance the study.

## 3. Results

### 3.1. Intersectoral Coordination Examples and Themes Discussed According to Each Strategic Line of Action of the PAHO One Health Policy

The participant countries presented their case studies, providing good examples of multisectoral communication, collaboration, coordination, and capacity building in zoonosis control, food safety, and antimicrobial resistance programs. Likewise, the three OHHLEP members (JCZ, NC, and NCB), presented the advances taken in place with the panel, such as the creation of the new One Health definition and existing tools to implement One Health action plans.

The consolidated country examples from each case discussed in the Brazil meeting are depicted in Table 1.

From the information gathered previously, the main outcomes identified were as follows:

- Early detection and reporting of zoonotic diseases in multiple countries (e.g., SARS-CoV-2 reports in animals in Argentina)
- Implementation of mandatory zoonosis notification and integrated surveillance systems (Argentina and Cuba)
- Establishment of multisectoral coordination groups and One Health committees/platforms (Belize, Brazil, Mexico, and Uruguay)
- Development and update of national policies on food safety and zoonosis control (Chile and Honduras)
- Capacity building through training webinars, educational materials, and technical groups (Brazil, Cuba, and Mexico)
- Regional cooperation and alignment with international One Health frameworks (Belize)
- Coordinated outbreak response and control of zoonotic diseases such as rabies, *Taenia solium*, and others (Belize, Cuba, Mexico, and Uruguay)

Challenges mentioned included the following: communication and coordination gaps between sectors and resource constraints, which countries are addressing with reforms and guides.

**Figure 1:**
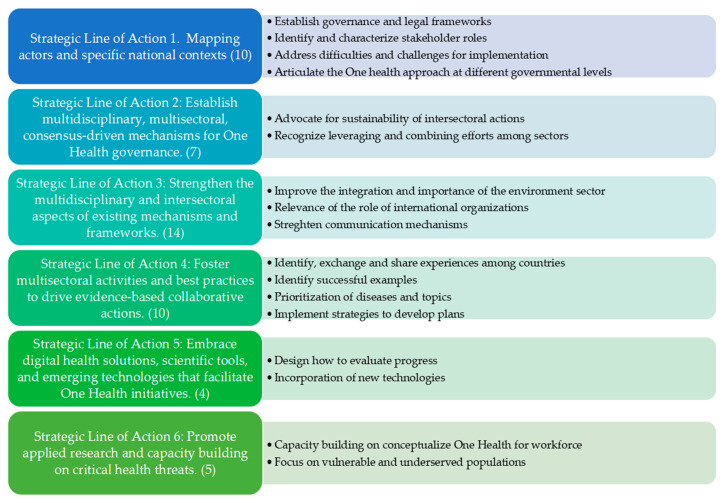
Diagram showing the results of the reflexive thematic analysis from the participant discussions according to the six strategic lines that make up PAHO’s One Health policy. In parentheses, the prevalence of the topics mentioned, and in the blue box to the right, the explanation of the codes that exemplify the theme.

The most important themes according to the strategic lines that make up PAHO’s One Health policy are presented in Figure 1. Topics were mainly grouped in the 6 strategic action lines as reflexive thematic analysis.

### 3.2. Lessons Learned from the Experiences of Latin America and the Caribbean Countries

After presentations and discussion based on the key questions, lessons learned emerged. We analyzed the content of the experiences presented by the representatives of the nine countries and identified the most important aspect of their learning. Then we socialized the results in the plenary and received their feedback. For instance, themes like “Collaboration and Communication” underscored the importance of joint problem-solving and cross-sector engagement, while “Harmonized Legislation” pointed to the need for updating laws to reflect the One Health perspective.

Additionally, “Equity” and “Cross-Sector and Cross-Region Learning” were emphasized to recognize inclusivity and knowledge exchange as essential factors for regional success. Analysis of themes also involved examining existing mechanisms, methodologies, and engagement strategies, as seen in themes like “Leverage Existing Communication Mechanisms” and “Use of Participatory Methodologies.”

Finally, emerging challenges and evolving contexts, including the impact of the COVID-19 pandemic and financing innovations, were addressed to frame a comprehensive picture of the current landscape and future directions.

**Figure 2:**
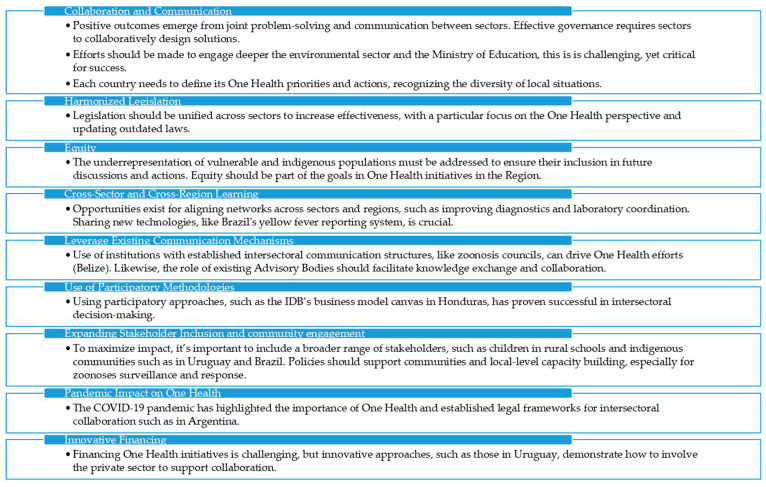
Scheme showing lessons learned from the experiences of focal points on zoonoses, antimicrobial resistance, and food safety in Latin American and the Caribbean countries extracted and categorized through consensus-building among facilitators.

The main results are summarized in Figure 2.

The lessons described by countries can be classified in the next topics: i. collaboration and communication, ii. harmonized legislation, iii. equity, iv. cross-sector and cross-regional learning, v. leverage existing communication mechanisms, vi. use of participatory methodologies, vii. expanding stakeholder inclusion and community engagement, viii. pandemic impact on One Health and ix. Innovative financing. The dialog was also focused on how countries have strengthened the risk analysis for the main food chains and the surveillance programs on antimicrobial resistance. The reflective shared learning among participants allowed an environment of trust that facilitated knowledge exchange, critical self-reflections, and positive feedback regarding the pitfalls and missteps among the different examples and experiences.

Some specific results to highlight from countries are as follows:

- In Honduras, the process leading to a shift toward a more integrated vision in the official Veterinary Public Health programs started with an internal reflection within organizations, which allowed the use of participatory methodologies for intersectoral decision-making, using the business model canvas.
- In Mexico, they started updating legislation regarding zoonoses and including the environment more clearly in the cooperation; they also include local knowledge and community engagement as a protagonist of change. Another key aspect developed was One Health prioritization of zoonoses using the USA Center for Disease Control (CDC) methodology and also starting with the implementation of a single program such as rabies, food safety, COVID, taeniasis.
- In Chile, the Chilean Agency for Food Safety and Quality (ACHIPIA) initiative, created advisory bodies that facilitate and promote change. They have not necessarily included the name One Health, but they have included risk analysis as an integrating axis in food safety programs. They highlighted the role of the private sector in financing One Health programs.
- In Uruguay they sought innovative financing to implement activities and potentiate the inclusion of the most relevant stakeholders at community level (children in rural schools and indigenous communities, etc.) to create more sustainable actions.
- In Belize, leaders use as drivers of change, the bodies within institutions that are already functioning and have functions or some type of intersectoral communication, such as zoonosis councils or committees. Interestingly, they valued personal relationships in building trust for joint cooperation.
- In Brazil, they strengthened epidemiological surveillance for infectious diseases and capacity building at community level as a common point in intersectoral cooperation.
- All countries have strengthened partnerships with academia and international cooperation, particularly regarding action plans such as antimicrobial resistance.

### 3.3. Examples of One Health Initiatives and Collaborative Networks Among Stakeholders in Latin America

In Table 2 we present the One Health initiatives and collaborative networks found on the internet from 2005 to 2024 in Latin American countries.

## 4. Discussion

### 4.1. Facilitators and Barriers in Multisectoral Collaboration

To the authors’ knowledge, this is the first reflective and participatory exercise of assessment and mapping of One Health initiatives from the Veterinary Public Health approach in Latin America and the Caribbean. This study identified the governmental examples of multisectoral collaboration in nine countries of Latin America and the Caribbean (Argentina, Belize, Bolivia, Brazil, Chile, Cuba, Honduras, Mexico, and Uruguay), their challenges, main outcomes, lessons learned, and areas of improvement. Simultaneously to the present study, another study on One Health priorities in Latin American countries has been conducted [4]. Both studies show that countries from Latin America and the Caribbean have been strengthening the One Health capacities and initiatives through the intersectoral work for more than seven decades. The main outcomes from the One Health initiatives across multiple countries highlight significant progress in zoonotic disease management and surveillance. Early detection and reporting systems have been strengthened. Mandatory zoonosis notification and integrated surveillance systems have been implemented in countries like Argentina and Cuba. Multisectoral coordination groups and One Health platforms have been established in Belize, Brazil, Mexico, and Uruguay, facilitating cross-sector collaboration. National policies on food safety and zoonosis control have been developed and updated, particularly in Chile and Honduras. Capacity building initiatives through training and educational resources have been conducted in Brazil, Cuba, and Mexico. Regional cooperation aligns countries with international One Health frameworks, and coordinated outbreak responses have effectively managed diseases such as rabies and *Taenia solium*. These achievements demonstrate enhanced intersectoral collaboration, surveillance, policy integration, and regional alignment to improve health security and address zoonotic threats comprehensively in Latin America.

This consolidated approach in the region, strengthens preparedness, response, and sustainable control of zoonoses, critical for public health resilience in the current threat of H5N1 Highly Pathogenic Avian Influenza. However, one area of improvement is the need to work more on strengthening the primary prevention focus, which is at the core of One Health priorities [9,10]. Effective primary prevention within the One Health framework emphasizes understanding the fundamental principles of disease emergence to thwart spillovers across human, animal, and environmental health sectors. It extends beyond traditional public health concerns, also prioritizing biodiversity conservation, environmental health, livestock production, food safety, and animal welfare [10]. We found that although there is a high-perceived importance on conjoint cooperation, the environmental component is not well-integrated into One Health actions, programs, and policies in Latin America and the Caribbean, despite the fact that the region has several countries listed among the 10 most mega-diverse countries in the world.

In our study, participants reported the most important barriers to implementing One Health interventions include weak governance, absence of regulatory frameworks, insufficient consensus on priorities. There is also a need for clearer guidelines on preparedness and response, as well as better formal collaboration and communication between sectors. Additionally, weaknesses in surveillance systems, limited understanding of One Health among sectors and communities, and a shortage of human, material, and logistical resources are significant obstacles. These findings correlate with key barriers reported by Dos Ribeiros in 2019 [11], stating the lack of political will, weak governance, and lack of human, financial, and logistical resources as main barriers. Likewise, in 2024, the European cross-agency One Health task force, pointed out that the lack of knowledge and awareness about One Health was one of the major limitations to operationalizing it at the national level [12]. In this sense, authors from the African context have expressed that significant challenges were as follows: limited diagnostic capacity for zoonotic diseases, hampered coordinated surveillance mechanisms, and a restrictive skilled workforce [13,14]. In the present study we found the same barriers found in the African context. On the other hand, we also found similarities with the study reported by Rai et al. 2025 [15]. They found that focused educational interventions, enhanced communication, strengthened collaboration, expanded research, and improved funding are essential for advancing One Health approaches and zoonotic disease prevention and control in Bhutan. Their findings, as well as our study, can inform global efforts to enhance One Health systems, particularly in regions where resources are limited but disease risks are significant [15].

In addition, social inequality was cited for participants as an important barrier because of the existing underrepresented vulnerable indigenous populations who must be included in early steps of projects, policies, and programs. Equity should be part of the goals in One Health initiatives in the region. Since the COVID-19 pandemic, there has been an increase in poverty rates, higher levels of unemployment, a deterioration in the quality of employment indicators, and stagnation in the reduction in income inequality [16,17]. The negative effects are more marked among the population living in poverty and precarious labor conditions, such as rural women, young people, indigenous people, Afro-descendants, migrants, small agricultural producers, and rural workers [18,19]. Therefore, in this context of inequity, the social determinants of health (SDH) should be seriously considered and taken into account in the discussion for the implementation of One Health in accordance with the underlying principles of One Health described in the OHHLEP definition [6,17]. Achieving these outcomes necessitates meaningful and inclusive engagement with all segments of the community—taking into account gender, indigeneity, age, and disability. Such engagement should actively seek and value local knowledge and lived experiences, and be grounded in respectful, trustworthy partnerships among communities, stakeholders, and implementing entities [20].

### 4.2. Best Practices and Gaps for One Health Implementation

The establishment of governance mechanisms and legal frameworks was the main concept discussed by participants in our workshop, followed by the importance and the need for inclusion of the environmental sector (Table 1 and Figure 2). Countries should focus on integrating the environmental sector better, which remains insufficiently connected within current One Health initiatives. This was pointed out in the survey carried out in 2021 [3], where participants stated that One Health can support countries to improve their health policies and health governance as well as to advocate the social, economic, and environmental sustainability of the region. In this sense, recently, some countries, such as Brazil and Cuba, have strengthened health systems through One Health frameworks and national strategies. In the case of Brazil, in the 1990s, with the creation of Brazil’s Unified Health System (Sistema Único de Saúde—SUS in Portuguese), based on a strong emphasis on primary health care, opportunities arose for various professionals, such as veterinarians, to become part of multidisciplinary teams, especially at the local level [21]. More recently, in 2024, the establishment of the Interinstitutional Technical Committee for One Health and the launch of the São Paulo Declaration on One Health are pivotal steps for the One Health implementation at the national level [22]. In the case of Cuba, the high-level governance process began in 2021 with the launch of the One Health Cuban National Strategy, and in 2025 the high-level political commitment facilitating intersectoral governance and the One Health mainstreaming in Cuba’s public policies has been remarkable. These two countries are setting an example to all countries in the Latin America and the Caribbean on the best practice of One Health Governance [23].

During the workshop, One Health best practices and gaps in PAHO One Health policy implementation were identified. These findings evidence the cumulative lessons learned by the countries after decades of making efforts for better surveillance, control, and prevention not only of endemic zoonoses, but also of emerging zoonoses such as SARS-CoV-2. The survey carried out by Rocha et al. 2024 [4]., identified that respondents face persisting difficulties in developing strategic roadmaps and governance mechanisms for implementing One Health nationally. Although Latin American and Caribbean countries show good alignment between national and international policies, indicating strong One Health performance globally, the integration between governments and communities remains a major challenge, particularly for local-level zoonosis management. Multiple obstacles such as creating feedback systems for implementing corrections, developing comprehensive monitoring and assessment frameworks, exchanging statistical methodologies, implementing standardized metrics for data evaluation, and guaranteeing system compatibility [4].

Participants in our present study, expressed their need to establish stronger governance and legal frameworks to facilitate the operative actions regarding the intersectoral work. These are the first steps described in the One Health Joint Plan of Action [8], as mapping the multisectoral coordination mechanisms (MCM) at the country level and establishing formal governance structures are best practices to pursue in the countries.

Our results show agreement with the results of the study carried out in 2017, the first survey aimed at presenting a baseline regarding priority zoonoses, capacities, and needs in Latin American and the Caribbean countries, they found that 39% of ministries indicated the need to create networks, 35% indicated the need to improve regional partnerships and integrate epidemiological surveillance processes, and, to a lesser extent, the need for integrated vector control, training of veterinarians and public health professionals, effective implementation of international regulations, standardization of updated programs on zoonotic diseases, and regular reporting of endemic and emerging zoonoses [24,25].

As in other regions of the world, the COVID-19 pandemic was also a key reason for strengthening One Health for zoonoses surveillance and prevention in Latin America and the Caribbean. It has exposed the absence of a comprehensive and efficient early warning system and early collaboration between stakeholders. Effective pandemic preparedness requires collaborative strategies and planning that bring together researchers, policymakers, the private sector, and all parties engaged in monitoring and early detection systems [26,27]. Veterinary Public Health is a ground that facilitates this transdisciplinary work, involving, a cultural shift toward cooperation to address complex, interconnected global health challenges such as emerging zoonotic diseases.

## 5. Conclusions

Veterinary Public Health is related to the cross-sectoral vision implicit in One Health, and a common thread of the holistic prevention required to face the challenges in global health, particularly in Latin America and the Caribbean. In this regard, for the Region of the Americas, the Pan American Center for Foot and Mouth Disease and Veterinary Public Health (PANAFTOSA/VPH) promotes technical-scientific cooperation for the development and strengthening of programs for the control and eradication of the main zoonoses, as well as intersectoral collaboration at the local and regional levels, with the objective of reducing the health, social, and economic impacts associated with the occurrence of zoonotic diseases. This analytical approach allowed for a structured synthesis of best practices and lessons learned, resulting in a thematic framework that guides decision-making and collaboration for effective One Health implementation.

One Health initiatives require shifting from a focus on individual disciplines to integrated, collaborative efforts that involve various sectors like human, animal, and environmental health, and include input from communities and policymakers. Learning from the existing initiatives presented in this study can exemplify and add value to other countries in the process of improving their practices and programs to achieve better results for human, animal, and environmental health. Strengthening One Health implementation in Latin America and the Caribbean requires enhancing governance mechanisms and legal frameworks to support effective intersectoral collaboration.

This assessment has provided an understanding of the history, efforts, challenges, and successes of the official zoonosis surveillance, prevention, and control programs. There is already installed capacity, a relevant knowledge and practice of the 4 C’s (communication, collaboration, coordination, and capacity-building) in the fields of VPH and, above all, multisectoral cooperation mechanisms that should be strengthened to better face future health emergencies within the Region.

PAHO and other international agencies and initiatives like the Inter-American Institute for Cooperation on Agriculture (IICA), WOAH, FAO, UNEP, OIRSA have strengthened competences and capacities around the prevention of zoonoses in many countries, creating a good baseline for One Health implementation in the American region.

## Figures and Tables

**Table 1:** Table summarizing intersectoral coordination examples reported by countries.

Country	Year/Period	Main Institution(s)	Key Actions/Initiatives
Argentina	2020–2022	Ministry of Health, CONICET, SENASA, Universities	Formation of an intersectoral working network. Developed joint guidelines on SARS-CoV-2 detection in animals, preventive measures in rescue centers and zoos, and wildlife management. Officially reported SARS-CoV-2 cases in domestic dogs and cats, as well as captive wild animals. In 2022, SARS-CoV-2 in animals became a mandatory notification event nationally.
Brazil	2019–2022	Ministry of Health, GT-Saúde Única	Created the One Health Technical Group (GT-Saúde Única) for coordination of zoonoses actions. Consultative group working on standardizing zoonoses surveillance using One Health. Public webinars and epidemiological bulletins on zoonoses and One Health. Discussion on creating inter-ministerial and institutional groups for zoonoses surveillance.
Chile	Since 2005	Ministry of Agriculture, Food Safety Agency	Created a Food Safety Agency (ACHIPIA) under Ministry of Agriculture with a council comprising multiple ministries. Developed and updated a National Food Safety Policy, promoting risk analysis, food quality, and international trade. Agency structured with areas for risk assessment, liaison with productive sector and academia, international affairs, and risk communication.
Cuba	2021	Ministries of Public Health and Agriculture	Surveillance and control of major zoonoses such as Rabies, West Nile Fever, Avian Influenza, Leptospirosis, and others. A new legal framework establishes collaboration between Ministries of Public Health and Agriculture. Developed leptospirosis vaccine requiring intersectoral cooperation. Updated avian influenza plan with specialists’ input in CENSA. Part of national technical working groups for One Health implementation.
Honduras	Recent	SENASA, Health and Environment Secretariats	Reformed SENASA’s structure and mission to incorporate One Health concepts. Reviewed processes and collaboration agreements, involving public health and environment secretariats. Developing a national integrated food control system as a project.
Mexico	Longstanding	Ministry of Health, CENAPRECE, SENASICA, Academia	Long history of controlling zoonoses like *Taenia solium* with epidemiological surveillance since the 1980s. National program uses One Health for prevention and control, aiming for elimination by 2024 per PAHO-WHO regional commitment. Strategy includes coordinated sampling and diagnosis in humans and swine, involving health authorities and academic institutions across multiple states.
Uruguay	Ongoing	Ministry of Public Health, Ministry of Livestock, Universities	National Honorary Zoonosis Commission (CNZ) comprising multiple ministries, universities, rural organizations, and veterinary and medical societies. CNZ uses multidisciplinary and transdisciplinary teams to tackle zoonoses including cystic echinococcosis. Provides integral vision and structural capacity embodying One Health principles.
Bolivia	Ongoing	SENASAG	Developing a national zoonotic diseases guide under One Health. Utilizing SENASAG structure and national zoonotic disease data to guide development.
Belize	Since 2013	Ministry of Health and Wellness, Belize Agricultural Health Authority, Ministry of Agriculture and Fisheries	Established Belize National One Health Committee. Coordinated multidisciplinary response to rabies outbreak, including investigations, managing human exposures with prophylaxis, and capture/removal of rabies-positive bats. Involvement in regional One Health policy ratification under CARICOM.

**Table 2:** Table showing the directory of initiatives and collaborative networks among several stakeholders in Latin America and the Caribbean.

**Name of the Initiative**	**Country/Location**	**Website/Social Media**
Sapuvetnet-OHIN	Latin America and Europe	https://www.sapuvetnet.org/ES_frameset.html accessed on 1 November 2025
One health Latinamérica Ibero y el Caribe—OHLAIC	Latin America–Ibero-America and the Caribbean	https://ohlaic.org/es/ accessed on 1 November 2025
South American Network of One Health—SANO	Latin America and the Caribbean	https://members.futureearth.org/page/lobby-home accessed on 1 November 2025
Comunidad de práctica de Una Salud	Latin America and the Caribbean	https://www.linkedin.com/groups/12940031/ accessed on 1 November 2025
Comunidad de Práctica de Una Salud y Biodiversidad	Latin America and the Caribbean	https://biodiversidadunasalud.com/index accessed on 1 November 2025
One Health Belize	Belize	https://onehealth.gov.bz/ accessed on 1 November 2025
One Health Brasil	Brazil	https://onehealthbrasil.com/ accessed on 1 November 2025
One Health Espirito Santo	Brazil	@one.health.es (Instagram) accessed on 1 November 2025
Associação Brasileira de Saúde Única	Brazil	https://abrasuni.com.br/ accessed on 1 November 2025
One Health Colombia	Colombia	https://www.facebook.com/OneHealthColombia/ accessed on 1 November 2025
One Health Student Club Salle	Colombia	@onehealthsalle (Instagram) accessed on 1 November 2025
One Health program (Fundación para la Conservación de la Biodiversidad Acuática y Terrestre (FUCOBI, Quito)	Ecuador	https://www.fucobi.org/una-salud.html accessed on 1 November 2025
Consorcio Colombia Wisconsin One Health	Colombia–US	https://webportal.gvn.org/node/18 accessed on 1 November 2025
Facultad Una Salud	Costa Rica	https://uci.ac.cr/es/oferta-academica accessed on 1 November 2025
Universidad de San Francisco de Quito	Ecuador	https://healthecuador.org/one-health/ accessed on 1 November 2025
Universidad Galileo	Guatemala	https://www.galileo.edu/facisa/carrera/diplomado-internacional-una-salud/ accessed on 1 November 2025
One Health Peru	Peru	https://www.facebook.com/onehealthperu @one.health.peru (Instagram) accessed on 1 November 2025
One Health Now	Chile	@onehealthnow_cl (Instagram) accessed on 1 November 2025
Una Salud Chile	Chile	@unasaludchile (Instagram) accessed on 1 November 2025
Instituto One Health de la Universidad Andres Bello	Chile	@onehealth.unab/ accessed on 1 November 2025
Una Salud Chile Argentina	Chile—Argentina	https://www.unasalud.cl/index.php accessed on 1 November 2025
One Health academy	Costa Rica	https://www.instagram.com/onehealthacademy_cr/ accessed on 1 November 2025 https://onehealthacademy.net/index.php/about-us/ accessed on 1 November 2025
Alianza One Health Selva Maya	Guatemala–Mexico	https://alianzaohselvamaya.info/ accessed on 1 November 2025
One Health Mx	Mexico	https://www.facebook.com/onehealthmx/ @onehealthmx/ (Instragram) accessed on 1 November 2025

## Data Availability

No new data were created or analyzed in this study. Data sharing is not applicable to this article.

## Abbreviations

The following abbreviations are used in this manuscript:

ACHIPIA	Agencia Chilena para la Inocuidad Alimentaria
AMR	Antimicrobial Resistance
CDC	Centers for Disease Control and Prevention
CNZ	Consejo Nacional de Zoonosis (Español)
CARICOM	Comunidad del Caribe
CENAPRECE	Centro Nacional de Control y Prevención de Enfermedades
CENSA	Centro Nacional de Sanidad Agropecuaria
CONICET	Consejo Nacional de Investigaciones Científicas y Técnicas
EMBRAPA	Empresa Brasileira de Pesquisa Agropecuária (Portuguese)
FAO	Food and Agriculture Organization
IICA	Inter-American institute for Cooperation on Agriculture
LAC	Latin America and The Caribbean
MCM	Multisectorial Coordination Mechanisms
OHHLEP	One Health High Level Expert Panel
OIRSA	Organismo Internacional Regional de Sanidad Agropecuaria
PAHO	Pan American Health Organization
PANAFTOSA/VPH-PAHO/WHO	Pan American Food and Mouth Disease and Veterinary Public Health Center, Pan American, Health Organization/World Health Organization (PANAFTOSA/VPH-PAHO/WHO)
REDIPRA	Reunión de Directores de los Programas de Rabia de las Américas
SENASA	Servicio Nacional de Sanidad e Inocuiadad Agropecuaria
SENASAG	Servicio Nacional de Sanidad Agropecuaria e Inocuidad Alimentaria
SENASICA	Servicio Nacional de Sanidad, Inocuidad y Calidad Agroalimentaria
SUS	Sistema Único de Saúde (Portuguese)
UNEP	United National Environmental Program
VPH	Veterinary Public Health
WOAH	World organization of Animal health
